# Supplementary material for: Upregulated Immunogenic Cell-Death-Associated Gene Signature Predicts Reduced Responsiveness to Immune-Checkpoint-Blockade Therapy and Poor Prognosis in High-Grade Gliomas
Source: Cells. 2022 Nov 17;11(22):3655. doi: 10.3390/cells11223655 (PMC9688114; doi:10.3390/cells11223655)
Supplement: Supplementary file 1 [file cells-11-03655-s001.zip › cells-1958748-supplementary/S1-2 figure legend.pdf]

Supplement Figure S1. Other ICD-associated identified subtypes in high-grade glioma. (A) Heatmap of consensus clustering (k = 2 ~ 9) for 33 genes. (B) Delta area curve and tracking plot of consensus clustering in area under the cumulative distribution function (CDF) curve for k = 2 ~ 9.

Supplement Figure S2. The survival of patients with the clinical prognostic characteristics respectively in TCGA and CGGA
