# Supplementary figures and images for: Upregulated Immunogenic Cell-Death-Associated Gene Signature Predicts Reduced Responsiveness to Immune-Checkpoint-Blockade Therapy and Poor Prognosis in High-Grade Gliomas
Source: Cells. 2022 Nov 17;11(22):3655. doi: 10.3390/cells11223655 (PMC9688114; doi:10.3390/cells11223655)

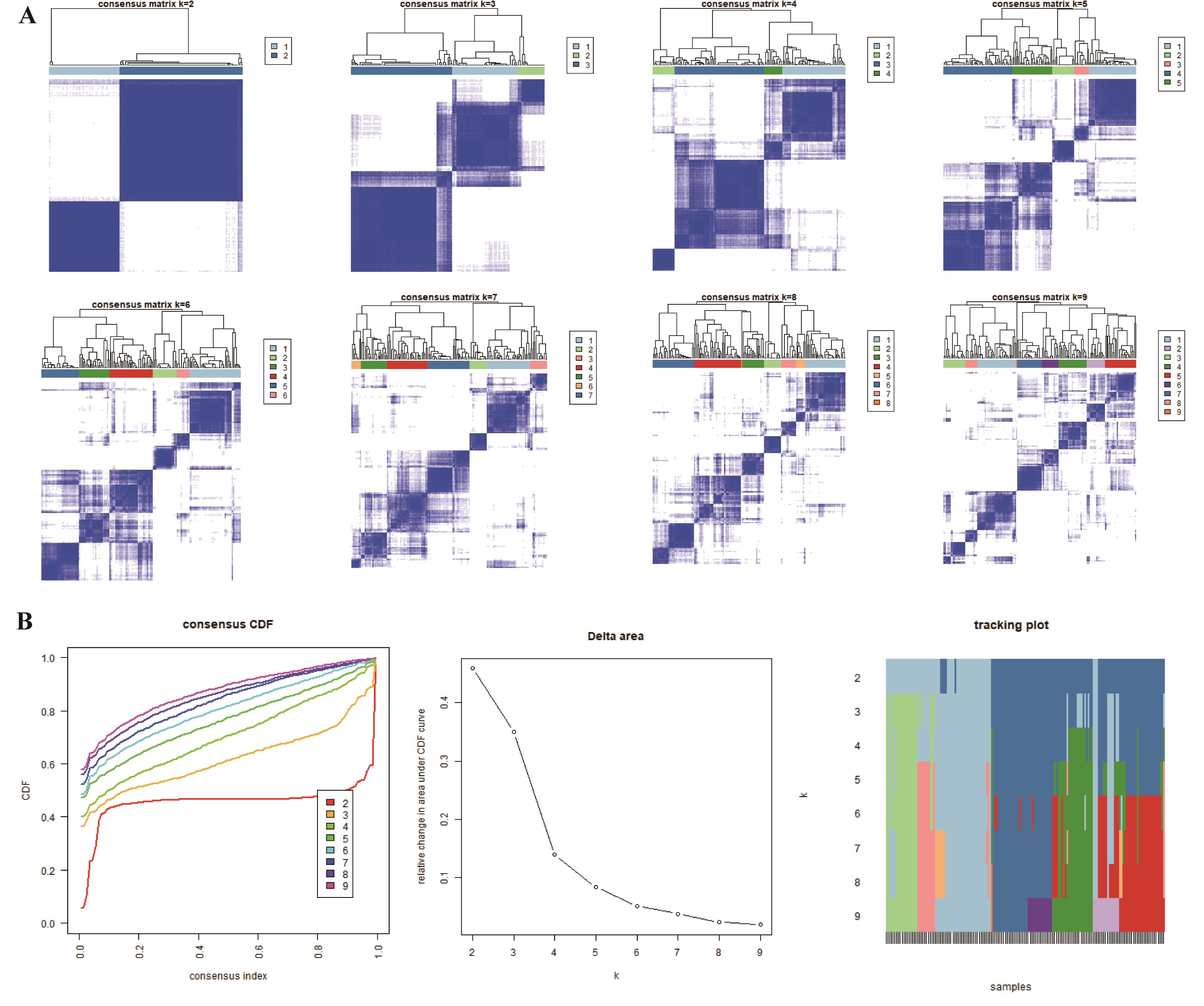

Supplement: Supplementary file 1 [file cells-11-03655-s001.zip › cells-1958748-supplementary/Supplement Figure S1.jpg]

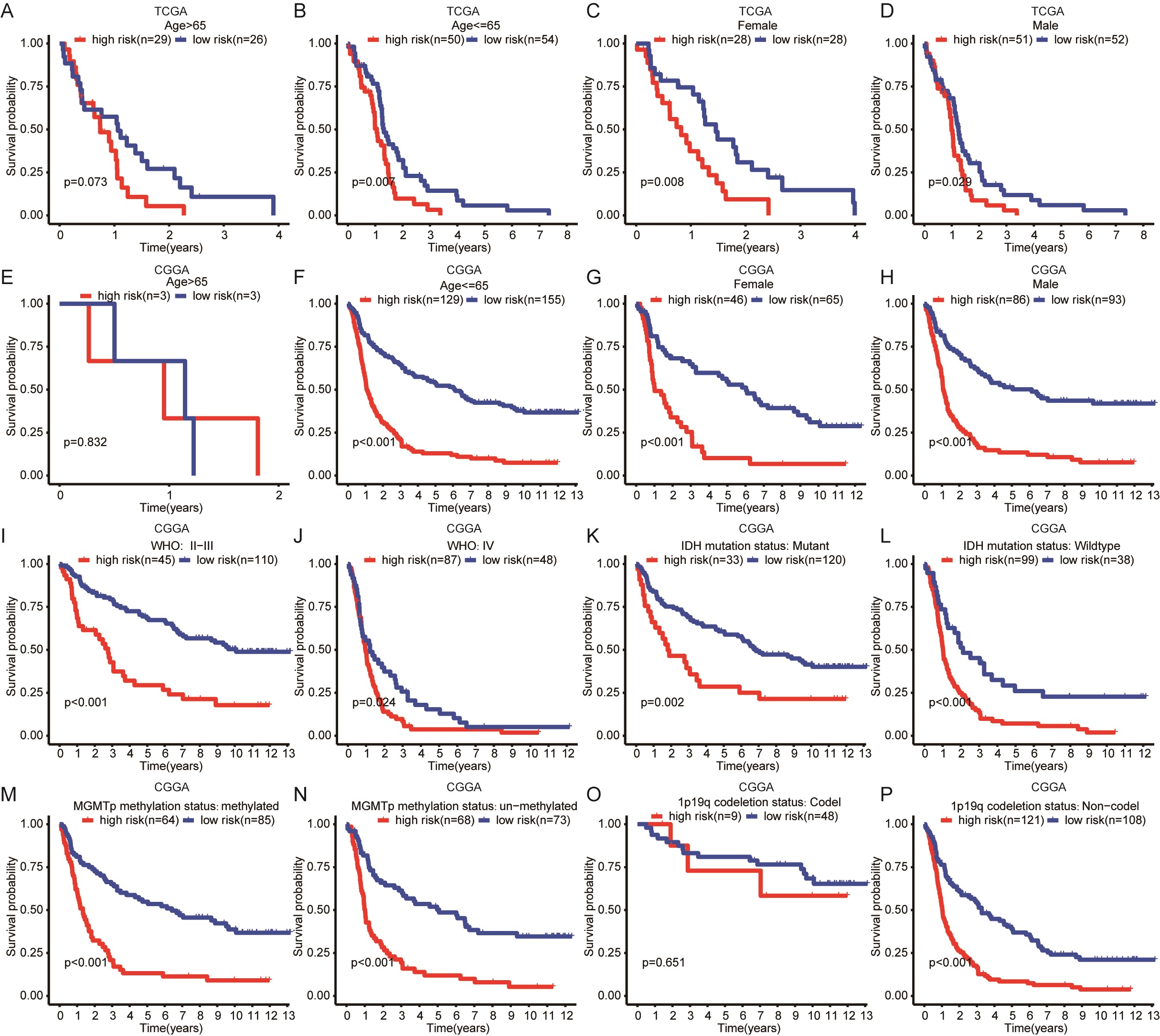

Supplement: Supplementary file 1 [file cells-11-03655-s001.zip › cells-1958748-supplementary/Supplement Figure S2.jpg]
